# Patients with Diabetes Experienced More Serious and Protracted Sickness from the COVID-19 Infection: A Prospective Study

**DOI:** 10.3390/medicina59030472

**Published:** 2023-02-27

**Authors:** Muiez Bashir, Wani Inzamam, Irfan Robbani, Tanveer Rasool Banday, Fahad A. Al-Misned, Hamed A. El-Serehy, Carmen Vladulescu

**Affiliations:** 1 Department of Radiodiagnosis and Imaging, SKIMS Soura, Srinagar 190011, India; 2 Department of Anaesthesia, SKIMS Soura, Srinagar 190011, India; 3 Department of Zoology, College of Science, King Saud University, Riyadh 11451, Saudi Arabia; 4 Department of Biology and Environmental Engineering, University of Craiova, 200585 Craiova, Romania

**Keywords:** COVID-19, RT-qPCR, HRCT, diabetes mellitus, computed tomographic severity score, immunocompromised

## Abstract

*Background and Objectives*: In December 2019, a flu-like illness began in the Chinese city of Wuhan. This sickness mainly affected the lungs, ranging from a minor respiratory tract infection to a severe lung involvement that mimicked the symptoms of Severe Acute Respiratory Syndrome (SARS). The World Health Organization (WHO) labelled this sickness as a pandemic in March 2020, after it quickly spread throughout the world population. It became clear, as the illness progressed, that people with concomitant illnesses, particularly diabetes mellitus (DM) and other immunocompromised states, were outmatched by this illness. This study was aimed to evaluate the correlation between Computed Tomographic Severity Score (CTSS) and underlying diabetes mellitus in coronavirus disease (COVID)-19 patients. *Materials and Methods*: This was a hospital-based prospective study in which a total of 152 patients with reverse transcriptase polymerase chain reaction (RT-PCR) positive COVID status who underwent high-resolution computed tomography (HRCT) of the chest were evaluated and categorized into mild, moderate and severe cases based on the extent of lung parenchymal involvement. A total score from 0–25 was given, based on the magnitude of lung involvement. Statistical analysis was used to derive a correlation between DM and CTSS, if any. *Results*: From our study, it was proven that patients with underlying diabetic status had more severe involvement of the lung as compared to non-diabetics, and it was found to be statistically significant (*p* = 0.024). *Conclusions*: On analysis of what we found based on the study, it can be concluded that patients with underlying diabetic status had a more prolonged and severe illness in comparison to non-diabetics, with higher CTSS in diabetics than in non-diabetics.

## 1. Introduction

In the Chinese city of Wuhan, the first instance of coronavirus disease 2019 (COVID-19), which was brought on by the SARS-CoV-2 coronavirus, was discovered in December 2019. The outbreak started in the Huanan seafood and livestock market in Wuhan, in the province of Hubei, and it gave some evidence of an animal to human transfer through the trade in seafood and live livestock. The virus quickly spread throughout the Chinese city of Wuhan and others by January 2020 [1]. It subsequently spread to other nations and continents, and in March 2020, the WHO proclaimed it to be a pandemic. It has been determined that the pathogen is a brand-new enveloped RNA coronavirus [2]. Electron microscopy has shown that the coronavirus is a spherical virus with a diameter of approximately 125 nm. These virions feature surface-based projections that resemble clubs. The coronavirus got its name because of these spikes, which make them resemble a solar corona. In the past, coronaviruses were believed to have a small involvement in human respiratory infections that were mild and self-limiting. Every year, 15–30% of respiratory tract infections are caused by endemic coronavirus infections in the human population [3]. The factors affecting the disease severity and mortality were still mainly unknown because it was a new ailment. The intensity of the symptoms is thought to be affected by several factors, including underlying health issues, patients over 65 years of age, and delayed hospitalization. A severe coronavirus infection is thought to be more likely to occur in patients with underlying medical conditions such as diabetes or high blood pressure. These patients also have a higher chance of dying from COVID-19 and are likely to be more vulnerable [4]. Despite the fact that COVID-19 has been linked to the dysfunction of numerous important organs, including the kidneys, liver, heart, brain, and gastrointestinal system, SARS-CoV-2 primarily targets the lung, and lung failure is the leading cause of death in COVID-19 patients. The respiratory epithelium is infected with SARS-CoV-2, which results in severe cough, increased mucus production, breathlessness, tightness of the chest, and wheezing. Pneumonia, the development of Acute Respiratory Distress Dyndrome (ARDS), and respiratory failure necessitating mechanical ventilation are the hallmarks of severe COVID-19 [5]. The severity of COVID-19 disease is exacerbated by the presence of underlying comorbidities. Moreover, these patients have a higher risk of hospitalization and an increased need for ICU admissions and ventilators. While they typically have the worst prognosis, individuals with infirmities should take all essential steps to prevent contracting SARS-CoV-2. These safety measures include avoiding close contact with others and practicing social distancing, regularly washing hands with soap and water or using an alcohol-based hand sanitizer, donning a face mask in public settings and avoiding being out in public unless absolutely essential [6]. Approximately 537 million individuals throughout the world have diabetes mellitus (DM), according to statistics [7]. India has been dubbed the “Diabetes Capital of the World” due to the high incidence of DM. In the Southeast Asian area, it is anticipated that the prevalence of diabetes will rise to 11.3% among people between the ages of 20 and 79. About 77 million Indian individuals had diabetes overall as of 2019, and by 2030, it is projected that this figure will increase to 100 million [8]. Patients with a CTSS of >18 have a greater probability of dying, and this score is a reliable predictor of mortality. Using an ideal CTSS cutoff in the visual scoring of lung involvement, the likelihood of mortality for COVID-19 patients could be precisely predicted [9]. The identification and continual evaluation of COVID-19 rely heavily on HRCT. In patients with COVID-19 pneumonia, the imaging findings include ground-glass opacities and/or consolidations. It has been determined that respiratory failure brought on by diffuse alveolar injury is the main cause of death. The production of hyaline membrane and severe inflammatory exudates in intra-alveolar spaces are correlated with the ground glass opacities. It was also discovered that people who died had severity scores that were greater than people who only had mild to moderate sickness [10]. China has been acknowledged as having made progress in the outbreak’s containment. The ability of China to effectively combat this disease is due to its robust economy, an abundance of economic resources, a strong government, efficient policy execution, and national cohesion. China took drastic measures to stop the spread of the disease by segregating suspected patients, keeping a tight eye on contacts, collecting data effectively, and developing diagnostic and treatment methods quickly and effectively. To contain the pandemic, enormous quarantine facilities were constructed and set aside [11].

This study aimed to establish an association between the severity of COVID-19 and DM.

## 2. Materials and Methods

### 2.1. Study Design

The study was a prospective one carried out in a tertiary care hospital, namely SKIMS Soura. IEC clearance was obtained. The IEC number of our study is IEC-SKIMS/2022-466.

### 2.2. Inclusion Criteria

All patients with RT-PCR proven COVID-19 status who had undergone HRCT chest scans. 

### 2.3. Exclusion Criteria

Patients with severe motion artefacts precluding CTSS assessment and those who had undergone contrast study.

### 2.4. Data Collection

The investigation of choice for diagnosis of COVID-19 is RT-PCR. Patients who had been diagnosed with COVID were subjected to HRCT chest scans. HRCT scans were performed between the 5th and 10th day after the onset of symptoms. Using 64-slice multidetector CT (SOMATOM, Siemens Health liners, Erlangen, Germany), HRCT chest scans were obtained with patients in the supine position and breath-holding. The following parameters were used: tube voltage of 120 kV, tube current of 100–200 mAs, collimation of 1.5–2.5 mm, and slice thickness of 1–2 mm. The HRCT chest scans were analyzed using lung and mediastinal window reconstruction algorithms. The data from the HRCT lung studies of 152 patients performed between March and June 2021, during the second wave of COVID-19, were prospectively collected and inspected to determine the connection between diabetes and COVID-19 severity. An experienced radiologist scrutinized the data to determine the CTSS of the COVID-19 lung infection. A numerical CT score was computed based on the grade of lung parenchymal involvement (range: 0–5 for each lobe; total score range: 0–25). CT severity score (CTSS), when computed, helps to classify patients into mild (0–11/25), moderate (12–18/25), and severe (>18/25) (Table 1). The lung lobar involvement was set side by side between diabetic and non-diabetic patients based on CTSS. 

### 2.5. Statistical Analysis

The categorical variables were presented in the form of numbers and percentages (%). On the other hand, the quantitative data with normal distribution were presented as the means ± SD, and the data with non-normal distribution as median with 25th and 75th percentiles (interquartile range). The data normality was checked by using the Kolmogorov–Smirnov test. For the cases in which the data were not normal, we used nonparametric tests. The following statistical tests were applied for the results: The comparisons of the variables that were quantitative and not normally distributed in nature were analyzed using the Kruskal–Wallis test, and variables that were quantitative and normally distributed in nature were analyzed using ANOVA. Univariate and multivariate logistic regression was used to identify significant risk factors for a moderate/severe CTSS (Table 2). Data entry was performed in Microsoft EXCEL spreadsheet, and the final analysis was conducted with the use of Statistical Package for Social Sciences (SPSS) software ver. 25.0 (IBM, Chicago, IL, USA). A p value of less than 0.05 was considered statistically significant.

## 3. Results

In our study, a total of 152 patients were enrolled. Out of these, 98 were males and 54 were females (approximately 65% males and 35% females, as depicted in Table 3). 

A total of 37 patients were diabetics (24.34%). Of these 152 patients, just one was below 20 years of age, and most of the patients were in the age group from 41–60 years, with a mean age of 53 years. Furthermore, in our study, 25 patients (16%) were or had a history of being smokers with varying pack years. Approximately 28 patients (18%) were on antihypertensives, although their blood pressure was under control. As many as five patients had chronic kidney disease (3.2%). Additionally, 12 patients were on inhalers for COPD (7.8%). Four patients were undergoing treatment for hematological malignancies (2.6%). In this study, three patients (1.9%) had incidental detection of lung masses, and two patients (1.3%) had lung nodules.

**Figure 1:**
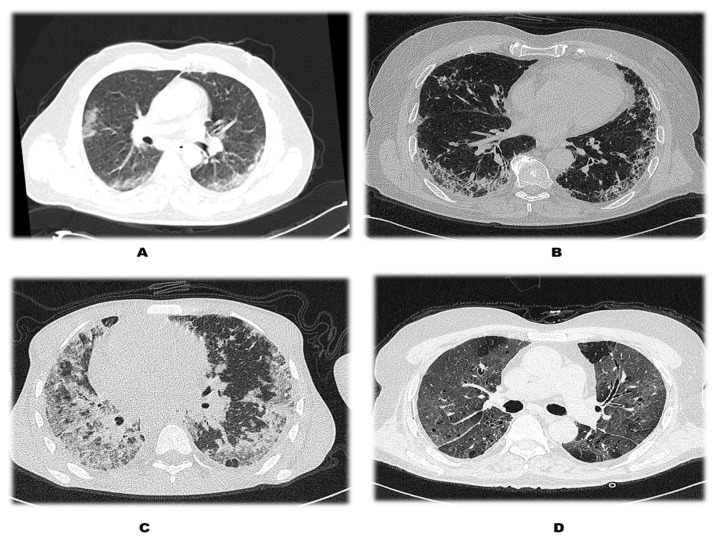
Pictorial representation of the severity of COVID-19 axial HRCT chest cuts with lung window demonstrating severity of disease: (**A**) Left upper—mild disease, (**B**) right upper—moderate disease, (**C**,**D**) severe disease.

Patients were labelled into mild (0–11), moderate (12–18), and severe (>18) categories based on the severity of lung involvement (Figure 1). 

Multivariate logistic regression was carried out to identify significant risk factors for moderate/severe CTSS (Table 4). 

Moderate and severe categories were classified into one category, and the association between DM and severe COVID-19 was calculated. Out of 152 patients with COVID-19, 77 (50.66%) had a mild score; 68 (44.74%) patients had a score of 12–18, which suggests moderate involvement, and the remaining 7 (4.61%) patients had a severe form of lung parenchymal involvement (Table 5).

**Figure 2:**
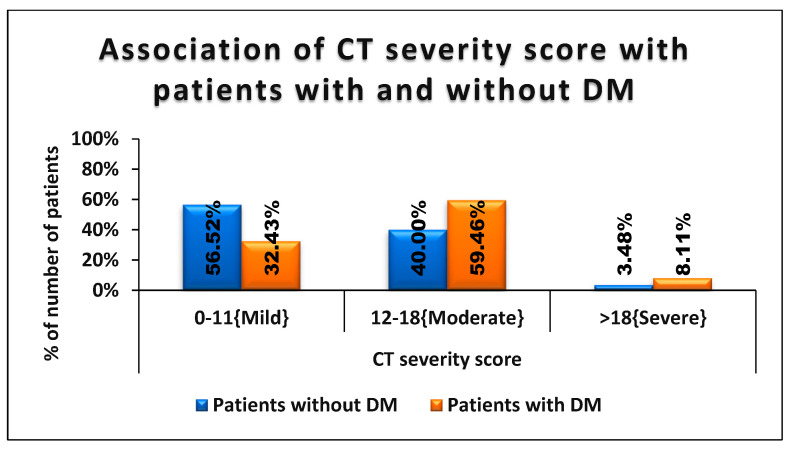
Association of CT severity score with patients with and without DM.

Of the patients without diabetes, 65 (56.62%) were affected by a mild form of COVID-19, 46 (40%) had moderate disease, and four (3.48%) had a severe form of lung involvement as per CTSS scores (Table 5). Of patients with diabetes, 22 (59.46%) had a moderate and three (8.11%) had severe form of the disease (Figure 2). This relationship between COVID-19 infection and serious lung involvement was statistically significant (*p* = 0.024).

**Figure 3:**
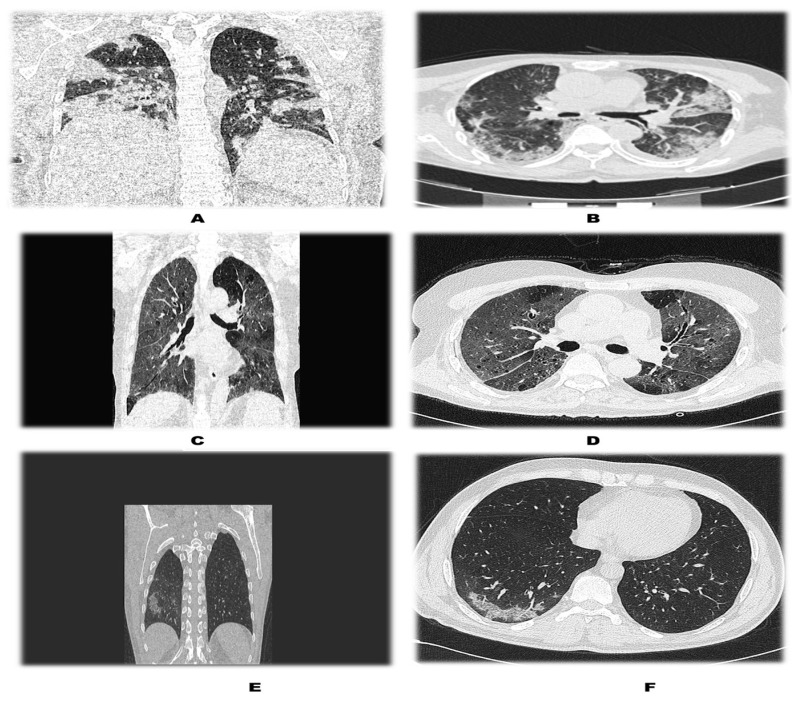
Axial and coronal cuts of patients with varying degrees of involvement of lung fields from severe to mild. (**A**,**B**) Coronal and axial cuts of a patient with severe involvement of the lung. (**C**,**D**) Coronal and axial cuts depicting moderate disease. (**E**,**F**) Mild involvement of lung field in a non-diabetic patient. The patient with severe lung involvement was the one who had underlying diabetes mellitus, clearly depicting severe involvement of lung fields.

Additionally, it was found that lung involvement in COVID-19 was more severe in diabetics than in non-diabetics (as depicted in Table 5). Axial and coronal CT images of the patients depicted grading of COVID-19 infection (Figure 3). 

Furthermore, it was found in our study that the mean duration of hospital stays of diabetics afflicted by COVID-19 was much higher than that of non-diabetics. We also noted longer recovery times for those COVID patients with underlying diabetes.

## 4. Discussion

As per the data currently available, it is proven beyond doubt that underlying diabetic status significantly affects the clinical outcome of COVID-19 patients. Diabetics who contract COVID-19 have worse prognosis and a higher risk of death, although it has been found that symptomatology between diabetics and non-diabetics does not vary much in patients with COVID-19. Diabetes and other chronic diseases continue to present one of the biggest problems for healthcare professionals in terms of meeting patient’s on-going needs. According to studies, preventing dysglycemia in diabetics can delay or even stop the development of their problems [12]. There is a high prevalence of diabetes in hospitalized COVID-19 patients. Moreover, underlying diabetes is a risk factor for higher mortality, especially in severely sick patients. Furthermore, it has been found that there is higher morbidity and mortality in diabetics who contract COVID-19 [13]. Diabetic patients with COVID-19 have a substantially higher mortality rate (15 vs. 2.3%) compared to COVID-19 patients without diabetes. Only a few research studies have demonstrated the link between diabetes and higher mortality risk (22–31% vs. 2–4%) [14]. The Computed Tomographic Severity Score (CTSS) is an indicator to gauge the severity of COVID-19 pneumonia [15]. Lower lobes of the lung, specifically the medial basal and lateral basal segments of the lung in both the right and left lower lobes, as well as the superior lingular segment, were the lobes most often afflicted [16].

Diabetic individuals showed a greater level of lung involvement than those without diabetes, which suggested a more serious lung infection, according to the quantifiable CT lung parenchymal involvement score [17]. A review of meta-analyses of various studies has shown that there is an association between COVID-19 and diabetic patients. The existence of chronic obstructive airway disease (COAD), acute kidney injury (AKI), underlying cardiac illness, advanced age, smoking and elevated BMI are additional variables that have been linked to poor outcomes in COVID-19 patients [18]. In a study conducted by the Chinese CDC, it was found that diabetics had a higher case fatality rate of 7.3%, in contrast to 2.3% in patients without diabetes. According to the Italian National Institutes of Health, 35.5% of patients who died from SARS-CoV-2 infection had diabetes [19]. Numerous other research studies have discovered that people with severe COVID-19 had a higher risk of developing diabetes, with some studies showing a two-fold increased risk. According to research by Wuhan Union Hospital using COVID-19 data, 21.2% of participants had diabetes, with radiographs showing advanced illness. Additionally, research from Italy similarly showed a 7.2% case fatality rate. Another study of ICU patients revealed that diabetes, hypertension, and cardiovascular disease were most frequently linked to serious illness [20].

There are clinically diagnosed stages of sickness in COVID-19 patients: the viremic stage, the acute stage, and the recovery stage. The joint action of the immune system’s innate and adaptive mechanisms is responsible for this clinical result. Viral infections are recognized by the innate immune system through toll-like receptors (TLRs). The generation of type I interferons and NK cell defensive mechanisms then occurs. The production of antibodies by the adaptive immune system limits the persistence of infection. Lymphopenia observed during the acute stage of COVID-19 disease is caused by CD4+ and CD8+ apoptosis. The percentage of CD4+ and CD8+ T-cells in patients with COVID-19 was much lower than it was in the control group, according to the results of immunophenotyping [21]. The poor clinical outcomes in patients with diabetes and cardiovascular diseases afflicted with COVID-19 appear to be due to the down-regulation of the ACE2 protein. Human ACE2 is mostly expressed in the small intestine, adipose tissue, kidneys, lungs, and nasal epithelium. High levels of ACE2 expression in nasal mucosa are consistent with clinical findings indicating symptomatic COVID-19 patients have higher viral loads in their nasal cavities than in their throats, which may explain why some patients report losing their sense of smell. The ectodomain of ACE2 is a dissolvable protein that, despite maintaining its catalytic function, can be discovered in blood in the circulatory system [22]. Without a shadow of a doubt, people with hypertension or over the age of 60 have greater needs for both invasive and non-invasive ventilation [23].

Onder et al. observed that out of 355 patients who died due to COVID-19 in Italy, diabetic status accounted for the most common comorbidity (35.5%) attributed to COVID-19 death, followed by cardiac ischemic diseases (30%) [24]. Wang G. et al. found higher chances of diabetes in those with severe disease (10.8% vs. 5.4%) [25]. In Wuhan, Wang D. et al. noticed that patients who were in need of ICU treatment (n = 36) were more likely to have diabetes than patients who did not require ICU care (n = 102) (22.2% vs. 5.9%) [26]. In their study, Wu et al. noted an increased incidence of SARS in COVID-19 patients who had underlying diabetic status than in non-diabetics. Furthermore, it was found that among those who had developed SARS, those that were diabetic were more likely to experience death. It is unclear how diabetes worsens COVID-19, but multiple factors may play a role [27]. NK cells are a crucial type of effector in innate immunity. The function of these cells is crucial for adaptive immunity. These cells can also activate or suppress T-cell responses. Ineffective T cell, NK cell and complement action causes inadequate viral clearance and hence may play a role [28]. Adaptive and innate immunity are influenced by dysglycemia in diabetics, which might explain the increased incidence of viral and bacterial infections involving the lung in these patients [29]. COVID-19, beyond doubt, has been proven to be a pro-inflammatory state and thus might aggravate the cytokine storm. It is regarded as the root cause of ARDS and systemic dysfunction [30]. Adipokines have been discovered to play a significant role in how diabetics who also have COVID-19 fare [31]. Given this, it is important to note that in Type 2 diabetes, aberrant adipokine and cytokine production, and reduced immunity are all associated with an increased risk of infection [32]. Increased SARS-CoV-2 pathogenicity, which has been connected to diabetes, has been linked to higher plasminogen levels. 

Diabetic patients are more likely to develop an aggressive form of coronavirus disease when their serum plasminogen levels are higher [33]. Serum levels of IL-6, ferritin, ESR, CRP, D-dimer and fibrinogen are significantly higher, as COVID-19 is an inflammatory and prothrombotic state [34]. An increase in ferritin, a protease enzyme associated with coronavirus entry into cells, may be the cause of increased viral replication in diabetes [35]. Patient care in diabetics should be improved to prevent morbidity issues and death [36]. For diabetics, self-care involves becoming aware of the complexity of their illness and acquiring the tools they need to manage it. Self-monitoring gives insight into one’s current glycemic state, enabling therapeutic evaluation and directing changes to one’s diet, exercise routine, and prescriptions in order to attain proper glycemic control [37]. Since the dysglycemia associated with diabetes is known to hinder patient recovery, diabetes and the COVID-19 condition may work in concert to adversely impair the patient’s prognosis. It is possible that COVID-19 and diabetes have a reciprocal connection, with SARS-CoV-2 either aggravating existing diabetes or causing those who are not diabetic to develop it. SARS-CoV-2 penetrates cells via the ACE2 pathway, and as ACE2 is extensively expressed in the liver and pancreas, it may have a role in insulin resistance and decreased insulin production. The genesis and evolution of human diabetes are mostly dependent on beta-cell dysfunction because of the cumulative effects of hereditary and acquired factors [38].

There is no doubt that the pancreas of people with diabetes has a lower number of islet cells or a reduced number of beta cells. A crucial component of appropriate and proper beta-cell function is normal beta-cell integrity. Diabetes patients have been shown to have a beta-cell mass of less than 60%. Although beta-cell mass is known to be significant, Type 2 diabetes mellitus etiopathogenesis is more strongly associated with beta-cell number [39]. To reiterate, SARS-CoV-2 penetrates cells via the ACE2 pathway, and as ACE2 is extensively expressed in the liver and pancreas, it may have a role in insulin resistance and decreased insulin production [40]. Increased oxidative damage and inflammation caused by high blood sugar levels have been seen to alter the expression of genes linked to aberrant insulin production and an increase in apoptosis. Damage to mitochondria, cell proteins, nucleic acids, and lipids results from oxidative stress. Additionally, all of this results in endoscopic reticulum stress, which in Type 2 diabetes mellitus causes beta-cell death. Pancreatic beta-cell infection in susceptible people may eventually result in beta-cell autoimmunity [41]. After the COVID-19 outbreak has passed, we might witness a rise in the prevalence of autoimmune diabetes. Although we are well-versed in the immediate concerns of COVID-19 patients, its long-term effects also demand vigilance. The length of stay in the hospital was prolonged in diabetics patients as compared to non-diabetics [42]. 

Diabetic patients have longer hospital stays and a higher incidence of complications. Numerous studies have conclusively shown that diabetics do have longer hospital stays and greater admission rates (2–6 times higher than non-diabetics). Hospitalization rates and length of stay have been observed to be correlated with serum levels of HbA1c, insulin demand, duration of diabetes, and presence of complications, and the worse these factors, the worse will be the outcome of the patient [43]. Although the increasing problems in diabetes are still not fully understood, the immune system may contribute to the worsening of the condition. Since IL-6 can trigger an immune response and the formation of effector T cells, it is known to defend against infections [44]. The synthesis of acute phase reactants such as serum amyloid A, fibrinogen, C-reactive protein (CRP), haptoglobin, and alpha 1-antichymotrypsin is induced by IL-6, which is first produced in a local lesion during the early stages of inflammation [45]. It is also known that IL-6 alters the control of iron absorption and increases hepatic hepcidin synthesis. Additionally, it has been discovered that IL-6 increases VEGF synthesis, which promotes angiogenesis and increases vascular permeability [46].

### Limitations

The limitations of our study were the limited sample size. In order to draw broader conclusions, further research including a larger sample size of individuals with complex clinical and laboratory correlations will be beneficial.

## 5. Conclusions

Our study has proven beyond doubt that diabetics are at escalated risk of developing a drastic form of COVID-19 illness. Any diabetic who has contracted COVID-19 should have strict glycemic control to avoid life-threatening complications thereof. Henceforth, diabetes is and should be considered one of the most important risk factors for developing a severe form of COVID-19 illness. Thus, the best way to reduce morbidity and mortality in diabetics is to avoid exposing them to COVID-19 positive patients. 

## Figures and Tables

**Table 1:** Tabular representation of grading of COVID-19 patients based on CTSS.

CLASS	CTSS (out of 25)
MILD	0–11
MODERATE	12–18
SEVERE	>18

**Table 2:** Univariate logistic regression to identify significant risk factors of moderate/severe CTSS.

Variable	Beta Coefficient	Standard Error	*p* Value	Odds Ratio	Odds Ratio Lower Bound (95%)	Odds Ratio Upper Bound (95%)
**Gender**						
Female				1.000		
Male	−0.040	0.339	0.905	0.960	0.494	1.866
**Diabetics**	0.973	0.397	0.014	2.645	1.214	5.761

**Table 3:** Distribution of demographic and baseline characteristics of study.

Demographic and Baseline Characteristics		
Age (Years)	Frequency	Percentage
≤20 years	01	0.66%
21–40 years	26	17.11%
41–60 years	86	56.58%
61–80 years	37	24.34%
>80 years	02	01.32%
**Mean ± SD**	52.9 ± 13.7	
Median (25th–75th percentile)	50 (45–61.25)	
Range	18–88	
**Gender**		
Female	54	35.53%
Male	98	64.47%
**Diabetics**	37	24.34%

**Table 4:** Multivariate logistic regression to identify significant risk factors for moderate/severe CTSS.

Variable	Beta Coefficient	Standard Error	*p* Value	Odds Ratio	Odds Ratio Lower Bound (95%)	Odds Ratio Upper Bound (95%)
Diabetics	0.886	0.425	0.037	2.426	1.054	5.584

**Table 5:** Association of CT severity score in patients with and without DM.

CT Severity Score	Patients without DM (n = 115)	Patients with DM (n = 37)	Total	*p* Value
0–11 {Mild}	65 (56.52%)	12 (32.43%)	77 (50.66%)	0.024
12–18 {Moderate}	46 (40%)	22 (59.46%)	68 (44.74%)	
>18 {Severe}	4 (3.48%)	3 (8.11%)	7 (4.61%)	
Total	115 (100%)	37 (100%)	152 (100%)	
